# Sickness absence and disability pension trajectories in childhood cancer survivors and references- a Swedish prospective cohort study

**DOI:** 10.1371/journal.pone.0265827

**Published:** 2022-04-01

**Authors:** Fredrik Baecklund, Kristina A. E. Alexanderson, Ellenor Mittendorfer-Rutz, Lingjing Chen

**Affiliations:** 1 Department of Microbiology, Tumor and Cell Biology, Karolinska Institutet, Stockholm, Sweden; 2 Pediatric Oncology Unit, Karolinska University Hospital, Stockholm, Sweden; 3 Division of Insurance Medicine, Department of Clinical Neuroscience, Karolinska Institutet, Stockholm, Sweden; Qatar University, QATAR

## Abstract

**Background:**

Childhood cancer survivors are at high risk of chronic health conditions. We aimed to explore future long-term sickness absence and disability pension in young adult childhood cancer survivors and matched references.

**Methods:**

We performed a prospective cohort study using microdata from five Swedish nationwide registers. Among all individuals born 1976–1998 and living in Sweden, we included 3632 childhood cancer survivors and 17,468 matched references that could be followed-up for 15, 10, or 5 years, respectively. A group-based trajectory model was applied to identify trajectories of mean annual sickness absence and/or disability pension days (SADP) in each sub-cohort, with 95% confidence intervals (CI). Potential risk factors for trajectory belonging were explored using χ^2^ test and multinomial logistic regression.

**Results:**

Most young adult childhood cancer survivors (90.2–96.5%) and references (97.4–98.8%) followed a No SADP trajectory. A larger proportion of childhood cancer survivors than references followed a Moderate (33–102 days/year) or High (115–260 days/year) SADP trajectory (15-year follow-up cohorts: Moderate 4.6% versus 1.2%; High 5.1% versus 1.5%). Childhood cancer survivors of central nervous system (CNS) tumors were at higher risk of the High SADP trajectory than childhood cancer survivors of hematological or non-CNS solid tumors (hematological versus CNS: odds ratio = 2.30, 95% CI 1.23–4.30; hematological versus non-CNS: odds ratio = 0.32, 95% CI 0.13–0.79).

**Conclusions:**

Although most young adult childhood cancer survivors had no SADP during follow-up, 9.7% experienced moderate or high numbers of SADP days/year throughout the 15-year follow-up; compared to 2.7% among references. CNS tumor survivors were at particular risk of SADP long-term and need extra attention in their future work prospect.

## Introduction

Survival after childhood cancer has improved substantially over the last decades and about 80% are alive five years after diagnosis [1, 2]. However, the cancer and its treatment (mainly chemotherapy, surgery, and/or radiotherapy) are associated with high risk of chronic health conditions among childhood cancer survivors (CCS), including cardiovascular diseases, endocrinopathies, renal impairment, mental disorders, and second cancers [3–7]. Such health conditions could potentially affect CCS’ need of sickness absence (SA) and disability pension (DP). This is a potential problem, because paid work provides a sense of normalcy and integration into society and improves overall health and wellbeing among adults, including CCS [8, 9].

In some previous studies, adult CCS were at higher risk of unemployment compared to a childhood cancer-free reference population [10–13], while this was not the case in other studies [14–17]. A higher risk of unemployment was found among CCS with a history of central nervous system (CNS) tumor [10–12, 14, 16, 17], young age at cancer diagnosis [11, 12, 15–17], female sex [11, 12, 15–17], and no/low educational level of the parents [11]. Furthermore, the risk of receiving social security benefits, including DP [10, 18, 19], early old-age retirement [17], and disability insurance [20] was higher among adult CCS than comparators. The risk was higher among adult CCS with a history of CNS tumor [10, 17–19] and young age at diagnosis [17, 20], but not female sex [10, 20].

These studies clearly suggest that young adult CCS have a higher risk of work absence, including SA and DP. However, considerable heterogeneity is expected, and there may be subgroups within the CCS population that have particularly high risk of SA and DP that are not detected using standard regression models [21]. Additionally, the need for social security benefits could be permanent or temporary, traits that are not captured in these previous studies. These issues may be solved by the use of group-based trajectory models [21, 22]. They allow for the identification of subgroups at particularly high risk of the outcome of interest and for graphical summaries of identified trajectories. Moreover, the use of both SA and DP as outcome measures provides a comprehensive picture of the burden of (chronic) health conditions among adult CCS, as SA implies temporal and DP long-term and permanent work disability.

The aim of this explorative study was to investigate future long-term SA and DP in young adult CCS and matched references, and to explore risk factors for trajectory-group belonging.

## Materials and methods

We conducted a prospective cohort study with matched references based on linked anonymized micro-data from five Swedish nationwide government-administered registers of high quality. The CCS and reference populations were identified among all individuals born 1976–1998 and who lived in Sweden at 20 years of age, according to the Swedish Total Population Register [23] (N = 3,225,285). Among them, 5777 individuals were diagnosed with cancer before aged 18 years (registered in the Swedish Cancer Register (24)) and had survived ≥5 years after diagnosis. Among the remaining 3.2 million individuals living in Sweden without cancer diagnosis at the starting year of follow-up (i.e., year of matching), five references were randomly selected and individually matched to each CCS by sex, birth year, birth country (Sweden or not), and parents’ highest educational level when CCS/reference person was aged 15.

The CCS and reference populations were then divided into three sub-cohorts each according to calendar period of diagnosis/matching and exact follow-up time available: 1) 1999–2003 (15-year follow-up), 2) 2004–2008 (10-year follow-up), and 3) 2009–2013 (5-year follow-up). Individuals with incomplete follow-up time (due to death or emigration) of 15, 10, or 5 years, respectively, were excluded. One reason for this was to capture all outcome events for each individual within a given time period, without any loss of follow-up. Another reason was that treatment protocols, cancer survival and health consequences may have changed over time. After applying these inclusion and exclusion criteria, 3632 CCS and 17,468 references remained and formed the study cohorts.

### Data sources

Information about the cancer diagnosis was obtained from the Swedish Cancer Register [24]. The coverage of newly detected tumors in this register is estimated to 96% and include data on age at diagnosis, diagnosis date, and type of cancer as International Classification of Disease version 7 (ICD-7) [25]. Cancer was categorized into three main categories and the most common subtypes using ICD-7 codes: 1) hematological malignancies, including lymphoma (200–202, 205–206) and leukemia (203–204, 207); 2) primary CNS tumor (193.0–193.2), and 3) non-CNS solid tumors, including neuroblastoma (193.3–193.9), retinoblastoma (192, 199.1), renal cancer (180), bone sarcoma (196), soft tissue sarcoma (197), germ cell tumors (175, 178), and others. Diagnosis age was categorized as 0–4, 5–9, 10–14, and 15–17 years and calendar period of diagnosis as 1979–1990, 1991–1995, 1996–2000, and 2001–2007.

The Statistics Sweden’ Longitudinal Integration Database for Health Insurance and Labor Market Studies (LISA) [26] covers all individuals aged ≥16 years since 1990, with data on birth country, and annual data on migration, educational attainment (elementary [≤9 years], high school [10–12 years], or university/college [>12 years]), and type of living area. The Micro Data for Analyses of Social Insurance (MiDAS) [27], kept by the Swedish Social Insurance Agency, includes detailed day-level data on SA and DP benefits for all in Sweden from 1994. The Cause of Death Register [28] was accessed for the date of death. Index person’s parents were identified from the Multi-generation Register [29] and their educational attainment from LISA.

### The public sickness absence and disability pension system in Sweden

All people in Sweden aged ≥16 years with income from work or unemployment benefits can be granted SA benefits if their work capacity is reduced due to disease or injury [30]. Because sick-pay for the first 14 days of a SA spell is provided by the employer, and thus not registered in MiDAS, we only included SA spells >14 days. All residents in Sweden aged 19–64 years with long-term or permanent work incapacity due to disease or injury can be granted DP. When aged 19–29, temporary DP can also be granted if morbidity necessitates more time to complete education. Both SA and DP can be granted at four levels of ordinary work hours (25%, 50%, 75%, or 100%). That means that people can be on both part-time SA and DP simultaneously. Therefore, we calculated net days for outcome measurement, combining SA and DP days. Two gross days of 50% benefits were, e.g., combined to one SADP net day (SADP day from here on). As there was no information in MiDAS on the first 14 days of the SA spells, they were calculated as having the same percentage as day 15 had. That is, if day 15 was for 25% of full-time, also the first 14 days were seen as being in 25%. If the SA spell was for only 15 days, that means 3.75 SA net days.

### Statistical analyses

Baseline sociodemographic characteristics and clinical covariates for the study population were categorized (details in Table 1) and their distributions calculated. A group-based trajectory model [31] was applied separately to each CCS and reference cohorts (defined by complete follow-up time; 15, 10, or 5 years) to identify distinct mean annual SADP days trajectories within each sub-cohort. That follow-up start at 20–23 for all was due to the requirement of ≥5-year survival after CC diagnosis (e.g., if diagnosed at 17 year, follow-up started at 23 years). We used the Bayesian Information Criterions to test and select the model of best fit related to the number of trajectory groups. Furthermore, the choice of trajectory groups was based on the proportions of individuals in every group being moderate.

**Table 1:** Characteristics of the study cohorts of young adult childhood cancer survivors and their matched references at start of follow-up.

Characteristics	Childhood cancer survivors (n = 3632)	Matched references (n = 17,468)
	n (%)	n (%)
***Socio-demographic factors***		
**Sex (women)**	1874 (51.6)	9029 (51.7)
**Age at the start of follow-up (years)**		
20	2783 (76.6)	13413 (76.8)
21	253 (7.0)	1216 (7.0)
22	284 (7.8)	1362 (7.8)
23	312 (8.6)	1477 (8.4)
**Start of follow-up period (duration of follow-up)**		
1999–2003 (15 years)	1036 (28.5)	4891 (28.0)
2004–2008 (10 years)	1140 (31.4)	5421 (31.0)
2009–2013 (5 years)	1456 (40.1)	7156 (41.0)
**Country of birth**		
Sweden	3483 (95.9)	16781 (96.1)
Outside of Sweden	149 (4.1)	687 (3.9)
**Parents’ highest education level when the index person was aged 15 years**		
Elementary	307 (8.5)	1468 (8.4)
High school	1729 (47.6)	8385 (48.0)
University/college	1596 (44.0)	7615 (43.6)
**CCS/references’ education level at start of follow-up**		
Elementary	1139 (31.4)	4166 (23.9)
High school	2329 (64.1)	12513 (71.6)
University/college	164 (4.5)	789 (4.5)
**Type of living area**		
Big cities	1332 (36.7)	6224 (35.6)
Urban	1577 (43.4)	7504 (43.0)
Rural places	723 (19.9)	3740 (21.4)
***Clinical characteristics***		
**Age at diagnosis (years)**		
0–4	1113 (30.6)	-
5–9	790 (21.8)	-
10–14	880 (24.2)	-
15–17	849 (23.4)	-
**Diagnosis period**		
1979–1990	880 (24.2)	-
1991–1995	1063 (29.3)	-
1996–2000	867 (23.9)	-
2001–2007	822 (22.6)	-
**Cancer diagnosis**		
**Hematological malignancies**	1385 (38.2)	-
Lymphoma	620 (17.1)	-
Leukemia	765 (21.1)	-
**CNS tumors**	871 (24.0)	-
**Non-CNS solid tumors**	1376 (37.8)	-
Neuroblastoma	100 (2.8)	-
Retinoblastoma	138 (3.8)	-
Renal tumors	185 (5.1)	-
Bone sarcomas	136 (3.7)	-
Soft tissue sarcomas	157 (4.3)	-
Germ cell tumors	206 (5.7)	-
Others	454 (12.5)	-
**Having sickness absence and/or disability pension in the start year of follow-up**		
Sickness absence (at least 3.75 net days in SA spells >14 days)	154 (4.2)	583 (3.3)
Disability pension (at least 7.5 days)	388 (10.7)	471 (2.7)
Sickness absence and/or disability pension	539 (14.8)	1050 (6.0)

CCS = childhood cancer survivors, CNS = central nervous system.

Thereafter, potential risk factors for belonging to each identified SADP trajectory group were explored among the CCS who completed the 15-year follow-up: sex, age at start of follow-up, age at CC diagnosis, birth country, diagnosis year category, parents’ highest educational level, index persons’ educational level follow-up start, and main cancer diagnosis categories (hematological, CNS, or non-CNS solid malignancy). Pearson’s χ^2^ test was applied to each potential risk factor, to evaluate if the proportions of CCS within each variable category differed between SADP trajectories more than would be expected by chance. The overall contribution of each potential risk factor to trajectory-group belonging was assessed by comparing two multivariable multinomial logistic regression models. The full regression model included all sociodemographic and clinical characteristics described above. The regression models for comparison were created by excluding the potential risk factor to be assessed from the full model. The difference in R^2^ between the full model and the model for comparison indicated the contribution of the excluded variable to the full model, and was tested for statistical significance using Nagelkerke pseudo R^2^ test. We further estimated the odds ratios (OR) with 95% confidence intervals (CI) of SADP trajectory-group belonging for each selected risk factors as using the full multinomial logistic regression model.

All statistical analyses were performed using SAS 9.4 (SAS-based procedure “Traj” [31], SAS Institute, Cary, NC). The significance level was set to 0.05.

The study was conducted in accordance with the World Medical Association Declaration of Helsinki [32]. Participant consent is generally not required in large register-based studies in the Nordic countries [33] and was waived by the Regional Ethical Review Board in Stockholm, Sweden, who approved of the project (approval numbers: 2007/762-31; 2009/23-32, 2009/1917-32; 2010/466-32; 2011/1710-32; 2011/806-32; 2016/1533-32). All data were anonymized by the administrative authorities before delivered to us.

## Results

Baseline characteristics of the CCS in 1979–2007 and their matched references are presented in Table 1. Around one fourth of diagnoses were primary CNS tumors. Most of the study population (77%) were followed from age 20, maximum age 23. Regarding education, 68.6% of CCS and 76.1% of references had at least some high school or university education at start of follow-up. The first year of follow-up, 14.8% of CCS and 6.0% of references had some SA (≥0.25 day) and/or DP (≥7.5 days).

**Fig 1:**
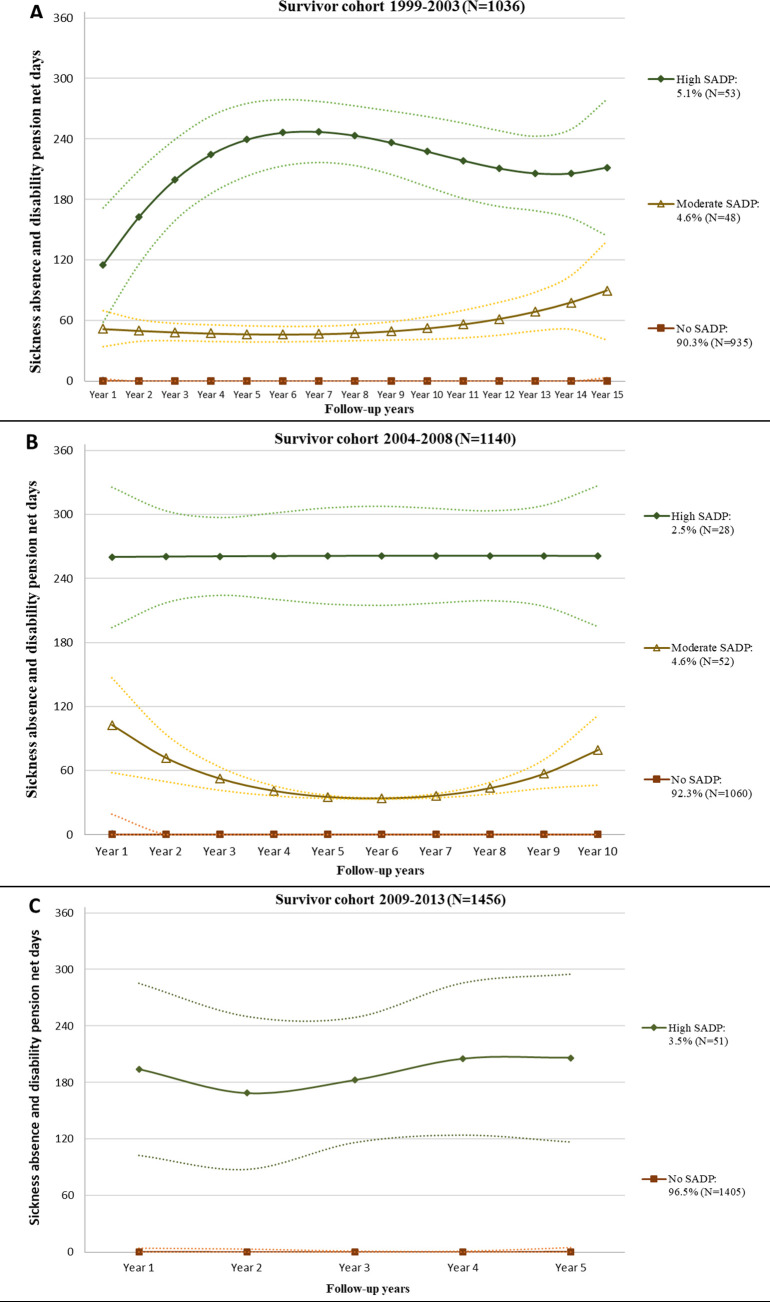
Estimated trajectories of annual mean sickness absence (SA) and disability pension (DP) days among childhood cancer survivors with 15 (A), 10 (B), or 5 (C) years of complete follow-up. Note: For each trajectory, the solid lines represent the predicted trajectory, and the broken lines represent the 95% confidence intervals. The legend indicates the number and percentage of the cohort belonging to each trajectory.

Among CCS and references with a 15- or 10-year follow-up, respectively, we identified three distinct trajectories of future SADP days/year, and among those who completed a 5-year follow-up we found two such trajectories (Fig 1 for CCS; S1 Fig for references). The dominant trajectory in all sub-populations was No SADP during follow-up. Among CCS with 15, 10, and 5 years of follow-up, the proportion following the No SADP trajectory was 90.2%, 93.0%, and 96.5%, respectively. Among the references, the corresponding proportions were higher: 97.4%, 97.9%, and 98.8%, respectively. Among CCS with 15-year (N = 1131) and 10-year (N = 1227) follow-ups, 53 (5.1%) and 28 (2.5%), respectively, followed a High SADP trajectory (115–260 days/year), while 48 (4.6%) and 52 (4.6%), respectively, followed a Moderate SADP trajectory (33–102 days/year). Among CCS completing only 5-year follow-up, 3.5% followed the High SADP trajectory. Only small proportions of the reference populations followed the High (15 years: 1.5%; 10 years: 1.0%; 5 years: 1.2%) or Moderate (15 years: 1.2%; 10 years: 1.1%) SADP trajectories.

The associations between potential risk factors and trajectory-group belonging among CCS completing 15 years of follow-up are presented in Table 2. The proportions of CCS within each category of the variables age at start of follow-up, parents’ educational level, CCS’ educational level, age at diagnosis, and type of cancer differed between trajectory groups more than would be expected by chance (Pearson’s χ^2^ test p<0.05). These five risk factors also contributed significantly to the full multinomial logistic regression model as estimated by the Nagelkerke pseudo R^2^. Survivors of CNS tumors compared to other tumor types (hematological versus CNS: OR = 2.30; 95% CI 1.23–4.30; hematological versus non-CNS solid: OR = 0.32; 0.13–0.79) and survivors with only elementary schooling compared to those with more education (OR = 3.01; 1.62–5.58) had higher risk of belonging to the High SADP trajectory after adjusting for all other sociodemographic and clinical factors.

**Table 2:** Distributions of sociodemographic and clinical characteristics, and their association with trajectory-group belonging among childhood cancer survivors with 15-year follow-up (N = 1036).

Characteristics	No SADP N (%)	Moderate SADP N (%)	High SADP N (%)	Pearson’s χ^2^	Nagelkerke R^2^ with variable removed	Diff in Nagelkerke to R^2^ full model	Multinomial regression OR (95% CI) No SADP versus Moderate SADP	Multinomial regression OR (95% CI) No SADP versus High SADP
**Total**	935 (100)	48 (100)	53 (100)				Full model	Full model
***Socio-demographic factors***								
Men	460 (49.2)	25 (52.1)	23 (43.4)	0.863	0.0792	0.0007	reference	reference
Women	475 (50.8)	23 (47.9)	30 (56.6)				1.30 (0.71 – 2.38)	0.92 (0.51 – 1.64)
**Age at the start of follow-up (years)**								
20	685 (73.3)	24 (50.0)	40 (75.5)	**17.8**	0.078	**0.0019**	reference	reference
21	70 (7.5)	10 (20.8)	4 (7.5)				2.07 (0.99 – 4.34)	1.05 (0.42 – 2.63)
22	79 (8.4)	5 (10.4)	5 (9.4)				0.97 (0.41 – 2.28)	1.44 (0.61 – 3.45)
23	101 (10.8)	9 (18.8)	4 (7.5)				1.40 (0.67 – 2.96)	0.85 (0.34 – 2.14)
**Country of birth**								
Sweden	902 (96.5)	45 (93.8)	51 (96.2)	0.958	0.0797	0.0002	reference	reference
Outside of Sweden	33 (3.5)	3 (6.3)	2 (3.8)				0.75 (0.18 – 3.11)	0.89 (0.18 – 4.35)
**Parents’ highest education level when the CCS was 15 years**								
University/college	372 (39.8)	20 (41.7)	16 (30.2)	**12.4**	0.0712	**0.0087**	reference	reference
High school	457 (48.9)	16 (33.3)	27 (50.9)				0.57 (0.29 – 1.14)	1.44 (0.74 – 2.79)
Elementary	106 (11.3)	12 (25.0)	10 (18.9)				1.88 (0.83 – 4.26)	2.14 (0.88 – 5.20)
**CCS education level at start of follow-up**								
At least high school	647 (69.2)	27 (56.2)	22 (41.5)	**21.7**	0.0582	**0.0217**	reference	reference
Elementary	288 (30.8)	21 (43.8)	31 (58.5)				**2.44 (1.29 – 4.64)**	**3.01 (1.62 – 5.58)**
**Type of living area**								
Big cities	324 (34.7)	17 (35.4)	20 (37.7)	0.256	0.079	0.0009	reference	reference
Urban	416 (44.5)	21 (43.8)	23 (43.4)				1.14 (0.57 – 2.27)	0.89 (0.46 – 1.71)
Rural places	195 (20.9)	10 (20.8)	10 (18.9)				1.34 (0.57 – 3.14)	0.75 (0.32 – 1.71)
***Clinical characteristics***								
**Age at diagnosis**								
0–4	242 (25.9)	7 (14.6)	20 (37.7)	**17.0**	0.073	**0.0069**	reference	reference
5–9	211 (22.6)	9 (18.8)	7 (13.2)				1.32 (0.47 – 3.74)	**0.31 (0.13 – 0.79)**
10–14	232 (24.8)	8 (16.7)	13 (24.5)				1.03 (0.18 – 5.69)	0.78 (0.21 – 2.94)
15–17	250 (26.7)	24 (50.0)	13 (24.5)				2.82 (0.73 – 10.9)	1.29 (0.40 – 4.14)
**Diagnosis period**								
1979–1990	449 (48.0)	16 (33.3)	29 (54.7)	7.59	0.0786	0.0013	reference	reference
1991–1995	345 (36.9)	19 (39.6)	16 (30.2)				0.97 (0.21 – 4.54)	0.55 (0.14 – 2.07)
1996–2000	141 (15.1)	13 (27.1)	8 (15.1)				1.24 (0.23 – 6.77)	0.70 (0.15 – 3.28)
**Cancer diagnosis**								
Hematological malignancies	372 (39.8)	22 (45.8)	21 (39.6)	**21.6**	0.0552	**0.0247**	reference	reference
CNS tumors	219 (23.4)	14 (29.2)	25 (47.2)				1.16 (0.57 – 2.39)	**2.30 (1.23 – 4.30)**
Non-CNS solid tumors	344 (36.8)	12 (25.0)	7 (13.2)				0.48 (0.22 – 1.01)	**0.32 (0.13 – 0.79)**

CCS = childhood cancer survivors, CNS = central nervous system, SADP = Combined sickness absence and disability pension net days. Note: the multinomial regression model estimated odds ratio (OR) with 95% confidence interval (CI) of belonging to Moderate or High SADP trajectories, given levels of sociodemographic and clinical characteristics. The full model included all variables included in this table.

## Discussion

In this register-based cohort study of 3632 CCS and 17,468 matched references followed 1999–2018, we identified three distinct SADP trajectories among the CCS and reference populations: No SADP, Moderate, and High SADP. The absolutely majority of both CCS and references belonged to the No SADP trajectories during follow-ups (90.2–96.5% of CCS and 97.4–98.8% of references). Reversely, larger proportions of CCS than references followed the Moderate (3.5–4.6% of CCS and 1.1–1.2% of references) and High trajectories (2.5–5.1% of CCS and 1.0–1.5% of references). Among CCS, risk factors for belonging to the High SADP trajectory were a history of CNS tumors and only elementary education.

In the St Jude Childhood Cancer Survivor Study from the USA, the prevalence of chronic health conditions of all grades was 77% already at 18 years of age and increased with time [3, 34]. Severe, disabling, and fatal chronic health conditions (grade 3–5) were observed in 38% at age 20–24 years and increased to 59% at age 35+ years [3, 34]. We did not have information about chronic health conditions in our CCS cohort. It is, however, reasonable to assume that it would be in the same range as in the St Jude CCS cohort, as treatments protocols used in Europe and USA are similar. Given the high prevalence of grade 3–5 health conditions already at age 20–24 years in the St Jude Study, the proportion of our CCS cohort following the Moderate and High SADP trajectories was rather low (up to 9.7%). Our results suggest that although the prevalence of health conditions may be high, the burden of them in terms of work incapacity was limited, since the vast majority of young adult CCS did not need SA or DP. This observation is in line with the one that most people with different diagnoses, the conditions do not imply such functional limitations that it affects their work capacity so much that they need SA or DP [35]. Furthermore, we found that a CCS aged 20–23 years with no SADP days is very likely to remain in this state for the next 15 years. This information is reassuring for most patients, parents, clinicians attending to children with cancer, and (future) employers.

Nevertheless, we found that up to 9.7% of CCS had a moderate to high mean number of SADP days/year already from the start of follow-up. Thereafter, their annual SADP either remained or increased during follow-up. Corresponding sub-cohorts were also identified among the references but the proportions were much smaller (1.2–2.7%). It is well documented that CCS are at higher risk of chronic health conditions than references without childhood cancer [3–7, 34]. Hence, a plausible reason for the observed differences between CCS and references in the current study may be late effects of the childhood cancer itself and/or its treatment.

Moreover, we found indications that childhood cancer type, level of parent’s and CCS own educational attainment, and age at diagnosis may be associated with risk of future SADP trajectory-group belonging. However, we only found a higher OR of High SADP trajectory among survivors of CNS tumor compared to other tumor types, and among survivors with only elementary schooling compared to others. Our results are in line with several studies demonstrating that childhood CNS tumor survivors have a particularly high risk of chronic health conditions [3], unemployment [10–12, 14–17], and receiving social security benefit [10, 17–20] compared to survivors of other CCs. Hence, this group of young adult CCS may need extra support to prepare for and to stay in the labor market. An association between low education and higher risk of SADP later in life is well described in non-CCS cohorts [36–38]. We speculate that the underlying cause may be different among CCS. Young adult CCS have a high prevalence of grade 3–5 chronic health conditions [3, 34] and a subgroup of CCS in the current study followed the Moderate and High SADP trajectories already from start of follow-up. This suggests that some CCS are affected by early onset chronic health conditions, likely caused by the tumor and/or its treatment. Such early onset chronic conditions may limit their opportunities of receiving higher education and taking paid work, potentially explaining the observed ORs in the current study.

A systematic review of determinants of socioeconomic outcomes in adult CCS found that those of younger age at diagnosis were at particular high risk of poor outcome, including DP, independent of cancer type [39]. Our findings, rather based on trajectory analyses than outcomes at a specific time period, do not support an association between age at diagnosis and such outcomes. Although the OR of High SADP trajectory belonging was higher among CCS diagnosed at age 0–4 years compared to those diagnosed when age 5–9 (OR = 0.31; 95% CI 0.13–0.79), there was no such risk increase compared to other age categories, nor between any age category and belonging to the Moderate SADP trajectory. Small numbers in each age category in the Moderate and High SADP trajectory groups makes our estimates uncertain, however, why young age at diagnosis as a risk factor for SADP cannot be excluded.

Among the strengths of this study was the use of high-quality micro-data from Swedish nationwide registers [23, 26] covering the whole population, which enabled the identification of virtually all CC patients during the study period, hence avoiding selection bias. Another advantage with using administrative registers, rather than specific research data or self-reported data, is that the data is collected independently of exposure and outcome status, reducing the risk of information and/or recall bias. Finally, for the first time in a population of CCS, we used a group-based trajectory model based on national administrative data on SADP benefits, which provides an illustration of the dynamics of the different patterns of future SADP days. In this way, the consequences of morbidity in terms of long-term work incapacity were studied. Given that the treatment of childhood cancer in Sweden follows international protocols, our results may be applicable to adult CCS in other countries with comparable healthcare and social insurance regulations.

Limitations with this study include the lack of treatment data, as type of treatment (e.g., use of radiotherapy, type and intensity of chemotherapy) is associated with the risk of chronic health conditions among CCS [3]. Thus, the impact of specific treatments on SADP trajectory-group belonging could not be investigated. However, we stratified the CCS cohort by diagnosis year, as a way to partly consider that treatment protocols may evolve over time. Inevitably, this led to different duration of follow-up periods for the three cohorts, which can be seen as a limitation in the comparisons. Moreover, information on SA spells shorter than 15 days was not available, which can be seen as both a limitation and a strength–in this way the outcome was not affected by short-term SA due to, e.g., trivial infections. Also, the lack of information on SA spells <15 days concerned both CCS and references.

## Conclusion

More than 90% of CCS did not have any SA or DP at age 20–23 years nor during follow-ups of 5–15 years. Although diagnoses indicating chronic health conditions are common among young adult CCS, this does not transfer to SA or DP in the vast majority. Nevertheless, an important minority of young adult CCS have moderate to high SADP already at follow-up start and throughout follow-up. CNS tumor and only elementary schooling were risk factors of high SADP levels. Further investigations are warranted to unravel which health conditions lie behind these SA and DPs in order to understand if further supportive measures can be taken to help affected CCS be part of the labor market.

## Supporting information

S1 Fig Estimated trajectories of annual sickness absence (SA) and disability pension (DP) days among references with 15, 10, or 5 years of exact follow-up. The Y axis represents net SADP days/year, the X axis follow-up time in years. For each trajectory, the solid lines represent the predicted trajectory, and the broken lines represent the 95% confidence intervals. The legend indicates the percentage of the cohort belonging to each trajectory.
(PDF)

S1 File (DOCX)
