# Supplementary material for: Sickness absence and disability pension trajectories in childhood cancer survivors and references- a Swedish prospective cohort study
Source: PLoS One. 2022 Apr 1;17(4):e0265827. doi: 10.1371/journal.pone.0265827 (PMC8975138; doi:10.1371/journal.pone.0265827)
Supplement: S1 Fig — The Y axis represents net SADP days/year, the X axis follow-up time in years. For each trajectory, the solid lines represent the predicted trajectory, and the broken lines represent the 95% confidence intervals. The legend indicates the percentage of the cohort belonging to each trajectory. (PDF) [file pone.0265827.s001.pdf]

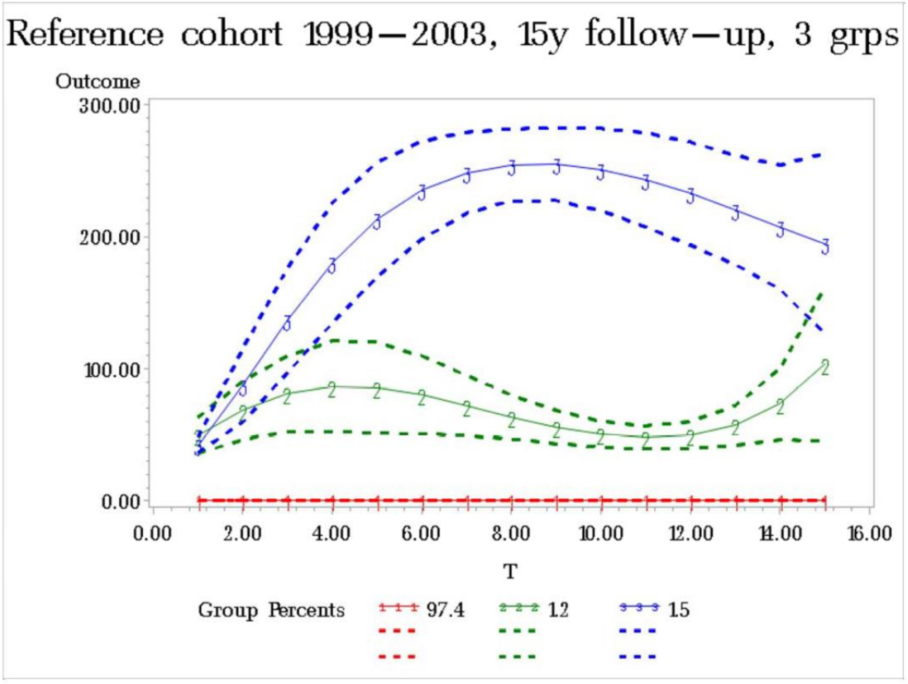
Reference cohort 1999−2003, 15y follow−up, 3 grps
Outcome
300.00
200.00
100.00
0.00
0.00
2.00
4.00
6.00
8.00
10.00
12.00
14.00
16.00
T
Group Percents
97.4
1.2
1.5

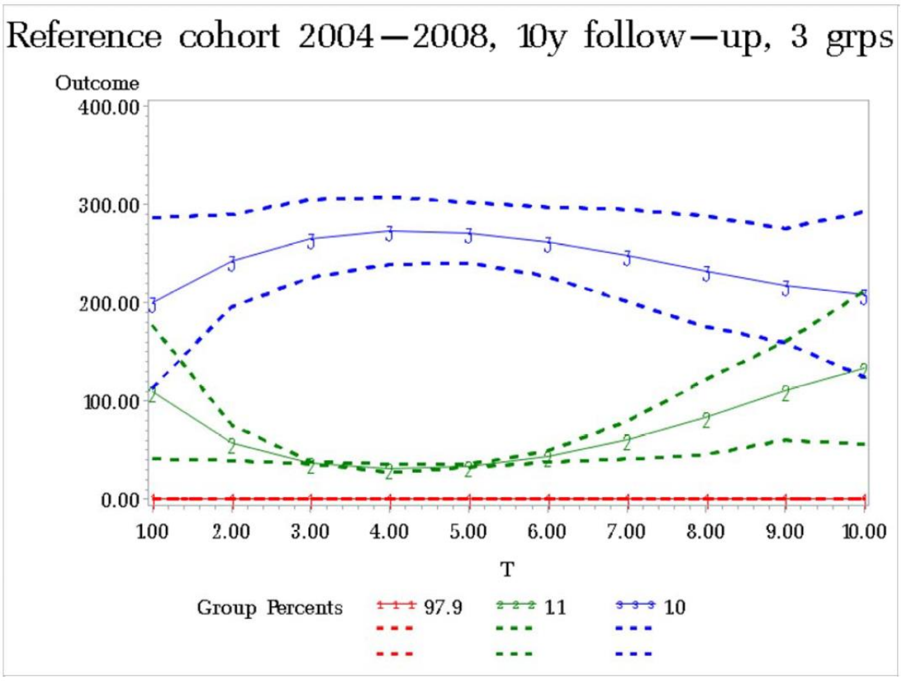
Reference cohort 2004−2008, 10y follow−up, 3 grps
Outcome
400.00
300.00
200.00
100.00
0.00
1.00
2.00
3.00
4.00
5.00
6.00
7.00
8.00
9.00
10.00
T
Group Percents
97.9
1.1
1.0

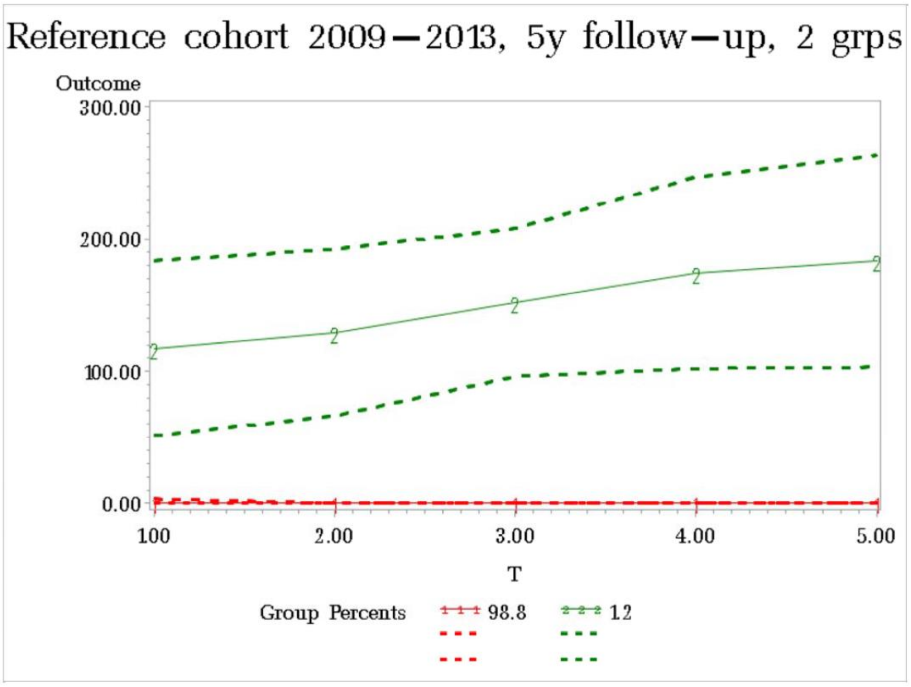
Reference cohort 2009−2013, 5y follow−up, 2 grps
Outcome
300.00
200.00
100.00
0.00
1.00
2.00
3.00
4.00
5.00
T
Group Percents
98.8
1.2
