# Supplementary material for: Sickness absence and disability pension trajectories in childhood cancer survivors and references- a Swedish prospective cohort study
Source: PLoS One. 2022 Apr 1;17(4):e0265827. doi: 10.1371/journal.pone.0265827 (PMC8975138; doi:10.1371/journal.pone.0265827)

**Supplementary results**

**Sickness absence and disability pension trajectories in childhood cancer survivors and references- a Swedish prospective cohort study**

**Authors**

Fredrik Baecklund MD PhD^1,2^; Kristina AE Alexanderson PhD^3^; Ellenor Mittendorfer-Rutz PhD^3^; Lingjing Chen MD MPH PhD^3^

**Author affiliation:**

^1^ Department of Microbiology, Tumor and Cell Biology, Karolinska Institutet, Stockholm, Sweden

^2^ Pediatric Oncology Unit, Karolinska University Hospital, Stockholm, Sweden

^3^ Division of Insurance Medicine, Department of Clinical Neuroscience, Karolinska Institutet, SE-171 77 Stockholm, Sweden

**Short title:**

Childhood cancer and future sick leave

**Supplementary table 1**. Distribution of demographic and clinical characteristics at start of follow-up among the childhood cancer survivors and references with 15 years of complete follow-up, by each of the three trajectory-group belongings.

|  | **Cancer survivors** | | | **References** | | |
| --- | --- | --- | --- | --- | --- | --- |
|  | **No SADP**  **N (%)** | **Moderate SADP**  **N (%)** | **High SADP**  **N (%)** | **No SADP**  **N (%)** | **Moderate SADP**  **N (%)** | **High SADP**  **N (%)** |
| **Total** | 935 | 48 | 53 | 4762 | 56 | 73 |
| **Sex** |  |  |  |  |  |  |
| Men | 475 (50.8) | 23 (47.9) | 30 (56.6) | 2443 (51.3) | 26 (46.4) | 24 (32.9) |
| Women | 460 (49.2) | 25 (52.1) | 23 (43.4) | 2319 (48.7) | 30 (53.6) | 49 (67.1) |
| **Age at start of follow-up (years)** | |  |  |  |  |  |
| 20 | 685 (73.3) | 24 (50.0) | 40 (75.5) | 3447 (72.4) | 28 (50.0) | 58 (79.5) |
| 21 | 70 (7.5) | 10 (20.8) | 4 (7.5) | 387 (8.1) | 6 (10.7) | 2 (2.7) |
| 22 | 79 (8.4) | 5 (10.4) | 5 (9.4) | 410 (8.6) | 9 (16.1) | 4 (5.5) |
| 23 | 101 (10.8) | 9 (18.8) | 4 (7.5) | 518 (10.9) | 13 (23.2) | 9 (12.3) |
| **Country of birth** |  |  |  |  |  |  |
| Sweden | 902 (96.5) | 45 (93.8) | 51 (96.2) | 4600 (96.6) | 53 (94.6) | 68 (93.2) |
| Other | 33 (3.5) | 3 (6.2) | 2 (3.8) | 162 (3.4) | 3 (5.4) | 5 (6.8) |
| **Parents education level when survivor/referent was 15 years old** | | | | |  |  |
| University | 372 (39.8) | 20 (41.7) | 16 (30.2) | 1848 (38.8) | 24 (42.9) | 18 (24.7) |
| High school | 457 (48.9) | 16 (33.3) | 27 (50.9) | 2319 (48.7) | 25 (44.6) | 44 (60.3) |
| Elementary | 106 (11.3) | 12 (25.0) | 10 (18.9) | 595 (12.5) | 7 (12.5) | 11 (15.1) |
| **Education level of survivor/referent at start of follow-up** | | | |  |  |  |
| At least high school | 647 (69.2) | 27 (56.2) | 22 (41.5) | 3569 (74.9) | 27 (48.2) | 23 (31.5) |
| Elementary | 288 (30.8) | 21 (43.8) | 31 (58.5) | 1193 (25.1) | 29 (51.8) | 50 (68.5) |
| **Type of living area** |  |  |  |  |  |  |
| Big cities | 324 (34.7) | 17 (35.4) | 20 (37.7) | 1656 (34.8) | 21 (37.5) | 28 (38.4) |
| Urban | 416 (44.5) | 21 (43.8) | 23 (43.4) | 2043 (42.9) | 28 (50.0) | 32 (43.8) |
| Rural | 195 (20.9) | 10 (20.8) | 10 (18.9) | 1063 (22.3) | 7 (12.5) | 13 (17.8) |
| **Age at diagnosis** |  |  |  |  |  |  |
| 0-4 | 242 (25.9) | 7 (14.6) | 20 (37.7) | - | - | - |
| 5-9 | 211 (22.6) | 9 (18.8) | 7 (13.2) |  | - | - |
| 10-14 | 232 (24.8) | 8 (16.7) | 13 (24.5) | - | - | - |
| 15-17 | 250 (26.7) | 24 (50.0) | 13 (24.5) | - | - | - |
| **Diagnosis period** |  |  |  |  |  |  |
| 1979-1990 | 449 (48.0) | 16 (33.3) | 29 (54.7) | - | - | - |
| 1991-1995 | 345 (36.9) | 19 (39.6) | 16 (30.2) | - | - | - |
| 1996-2000 | 141 (15.1) | 13 (27.1) | 8 (15.1) | - | - | - |
| **Cancer diagnosis** |  |  |  |  |  |  |
| Hematological | 372 (39.8) | 22 (45.8) | 21 (39.6) | - | - | - |
| CNS | 219 (23.4) | 14 (29.2) | 25 (47.2) | - | - | - |
| Non-CNS | 344 (36.8) | 12 (25.0) | 7 (13.2) | - | - | - |

SADP= Combined sickness absence and disability pension net days

**Supplementary table 2**. The odds ratio (OR) with 95% confidence intervals (CI) of Moderate or High SADP trajectory, given levels of sociodemographic and clinical characteristics (predictors) among the childhood cancer survivors with 15 years of complete follow-up. The adjusted multinomial regression model included all variables listed in the table. Significant associations (P <0.05) in bold.

|  | **Cancer survivors (n = 1036 )** | | | |
| --- | --- | --- | --- | --- |
|  | **Unadjusted** | | **Adjusted** | |
|  | OR (95% CI) | OR (95% CI) | OR (95% CI) | OR (95% CI) |
| Predictors | Moderate SADP  (n = 48) | High SADP  (n = 53) | Moderate SADP  (n = 48) | High SADP  (n = 53) |
| **Sex** |  |  |  |  |
| Men | reference | reference | reference | reference |
| Women | 1.12 (0.63 – 2.01) | 0.79 (0.45 – 1.38) | 1.30 (0.71 – 2.38) | 0.92 (0.51 – 1.64) |
| **Age at start of follow-up (years)** | |  |  |  |
| 20 | reference | reference | reference | reference |
| 21 | **4.08 (1.87 – 8.87)** | 0.98 (0.34 – 2.82) | 2.07 (0.99 – 4.34) | 1.05 (0.42 – 2.63) |
| 22 | 1.81 (0.67 – 4.87) | 1.08 (0.42 – 2.83) | 0.97 (0.41 – 2.28) | 1.44 (0.61 – 3.45) |
| 23 | **2.54 (1.15 – 5.63)** | 0.68 (0.24 – 1.94) | 1.40 (0.67 – 2.96) | 0.85 (0.34 – 2.14) |
| **Country of birth** |  |  |  |  |
| Sweden | reference | reference | reference | reference |
| Other | 1.82 (0.54 – 6.17) | 1.07 (0.25 – 4.59) | 0.75 (0.18 – 3.11) | 0.89 (0.18 – 4.35) |
| **Parents education level when survivor was 15 years old** | | |  |  |
| University | reference | reference | reference | reference |
| High school | 0.65 (0.33 – 1.27) | 1.37 (0.73 – 2.59) | 0.57 (0.29 – 1.14) | 1.44 (0.74 – 2.79) |
| Elementary | 2.11 (1.00 – 4.45) | 2.19 (0.97 – 4.98) | 1.88 (0.83 – 4.26) | 2.14 (0.88 – 5.20) |
| **Education level of survivor at start of follow-up** | | |  |  |
| At least high school | reference | reference | reference | reference |
| Elementary | 1.75 (0.97 – 3.14) | **3.16 (1.80 – 5.56)** | **2.44 (1.29 – 4.64)** | **3.01 (1.62 – 5.58)** |
| **Region** |  |  |  |  |
| Big cities | reference | reference | reference | reference |
| Urban | 0.96 (0.50 – 1.85) | 0.90 (0.48 – 1.66) | 1.14 (0.57 – 2.27) | 0.89 (0.46 – 1.71) |
| Rural | 0.98 (0.44 – 2.18) | 0.83 (0.38 – 1.81) | 1.34 (0.57 – 3.14) | 0.75 (0.32 – 1.71) |
| **Diagnosis age** |  |  |  |  |
| 0-4 | reference | reference | reference | reference |
| 5-9 | 1.47 (0.54 – 4.03) | **0.40 (0.17 – 0.97)** | 1.32 (0.47 – 3.74) | **0.31 (0.13 – 0.79)** |
| 10-14 | 1.19 (0.43 – 3.34) | 0.68 (0.33 – 1.39) | 1.03 (0.18 – 5.69) | 0.78 (0.21 – 2.94) |
| 15-17 | **3.32 (1.40 – 7.85)** | 0.63 (0.31 – 1.29) | 2.82 (0.73 – 10.93) | 1.29 (0.40 – 4.14) |
| **Diagnosis year** |  |  |  |  |
| 1979-1990 | reference | reference | reference | reference |
| 1991-1995 | 1.55 (0.78 – 3.05) | 0.72 (0.38 – 1.34) | 0.97 (0.21 – 4.54) | 0.55 (0.14 – 2.07) |
| 1996-2000 | **2.59 (1.21 – 5.51)** | 0.88 (0.39 – 1.97) | 1.24 (0.23 – 6.77) | 0.70 (0.15 – 3.28) |
| **Cancer type** |  |  |  |  |
| Hematological | reference | reference | reference | reference |
| CNS | 1.08 (0.54 – 2.16) | **2.02 (1.11 – 3.70)** | 1.16 (0.57 – 2.39) | **2.30 (1.23 – 4.30)** |
| Non-CNS | 0.59 (0.29 – 1.21) | **0.36 (0.15 – 0.86)** | 0.48 (0.22 – 1.01) | **0.32 (0.13 – 0.79)** |

SADP= Combined sickness absence and disability pension net days, CNS= central nervous system

**Supplementary figure 1**. Estimated trajectories of annual sickness absence (SA) and disability pension (DP) days among references with 15, 10, or 5 years of exact follow-up. The Y axis represents net SADP days/year, the X axis follow-up time in years. For each trajectory, the solid lines represent the predicted trajectory, and the broken lines represent the 95% confidence intervals. The legend indicates the percentage of the cohort belonging to each trajectory.


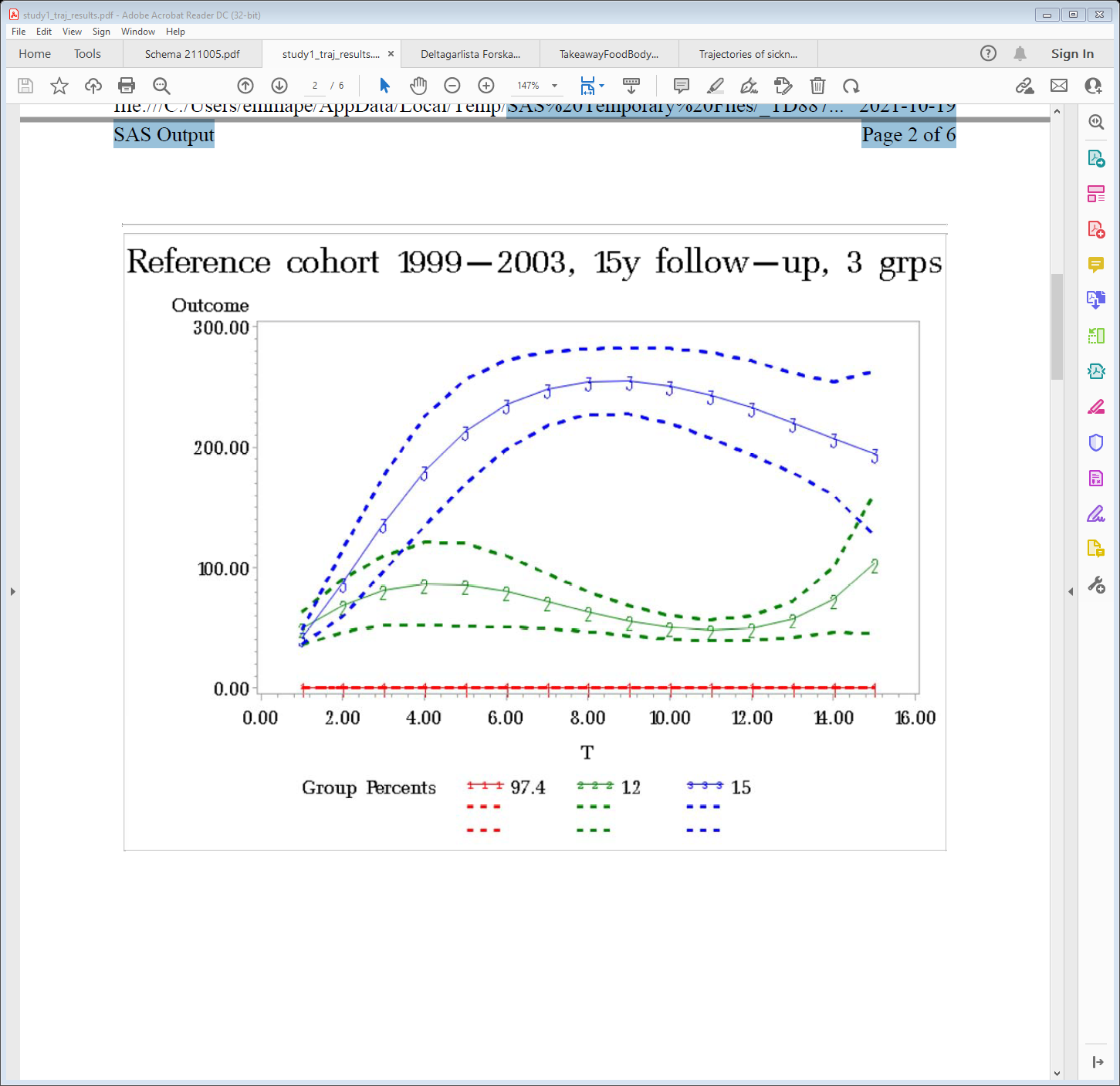


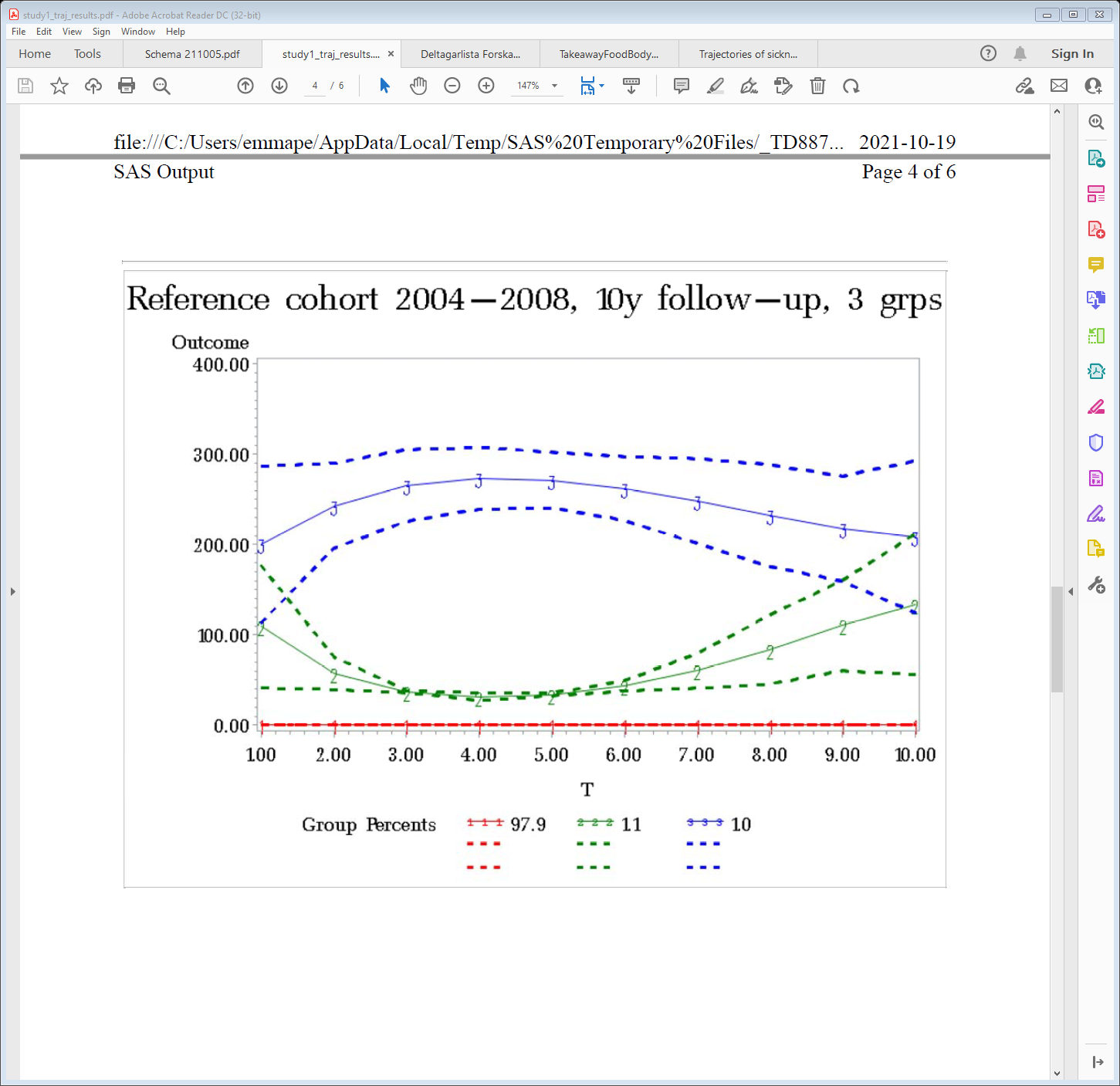


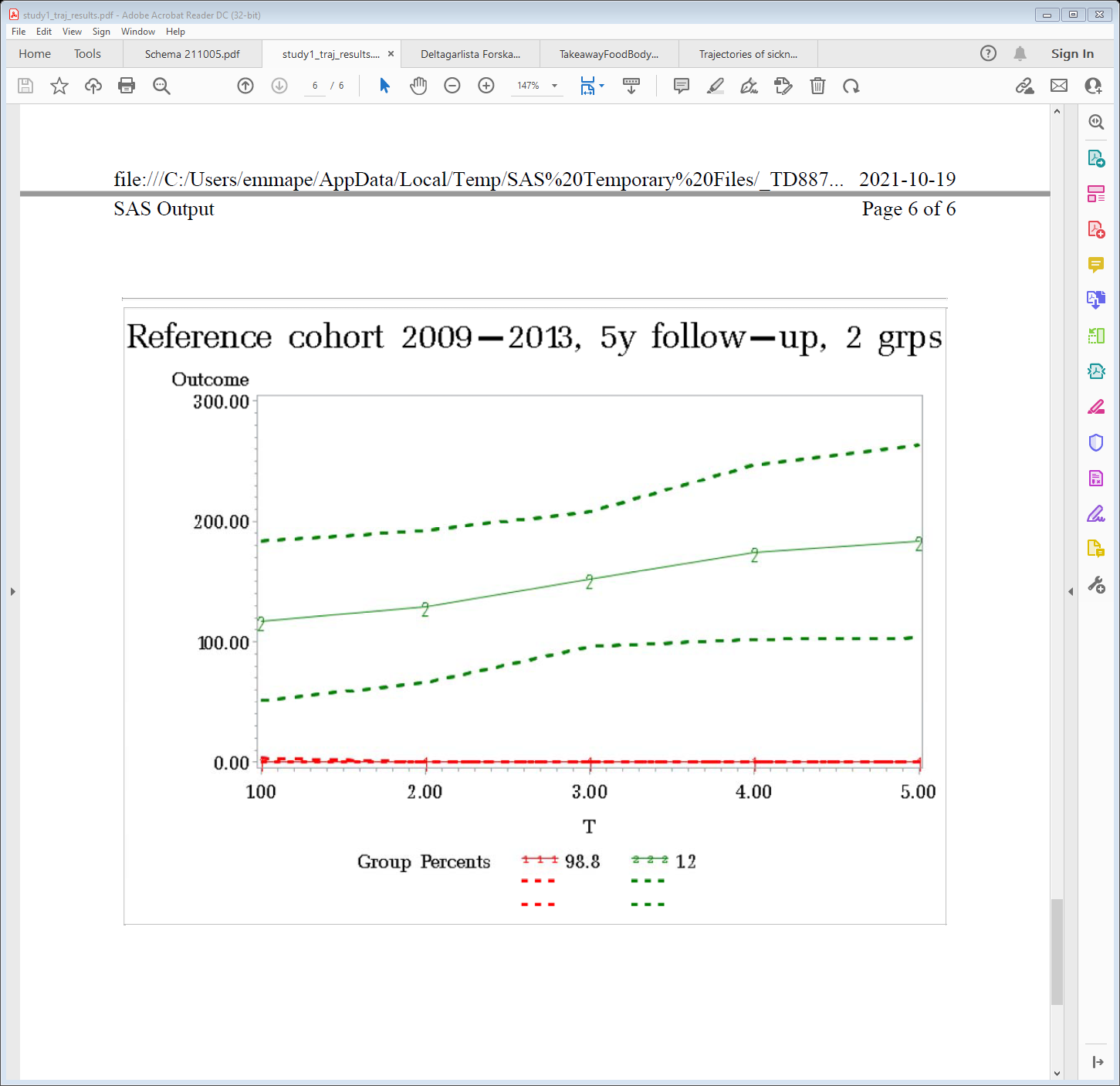

Supplement: S1 File — (DOCX) [file pone.0265827.s002.docx]
